# G9a inhibition potentiates the anti-tumour activity of DNA double-strand break inducing agents by impairing DNA repair independent of p53 status

**DOI:** 10.1016/j.canlet.2016.07.009

**Published:** 2016-10-01

**Authors:** Pallavi Agarwal, Stephen P. Jackson

**Affiliations:** a The Wellcome Trust/Cancer Research UK Gurdon Institute and Department of Biochemistry, University of Cambridge, Cambridge CB2 1QN, UK; b The Wellcome Trust Sanger Institute, Hinxton, Cambridge CB10 1SA, UK

**Keywords:** Cancer epigenetics, Chemical probes, UNC0638, Chemotherapeutics, Non-homologous end joining, DSB, double strand break, NHEJ, non-homologous end joining, SGC, Structural Genomics Consortium, DDR, DNA damage response, IC50, inhibitory concentration 50%, WT, wild-type, KO, knockout, PI, propidium iodide, XRCC4, X-ray repair cross-complementing protein 4, ATM, ataxia telangiectasia mutated, ATR, ataxia telangiectasia and Rad3-related protein

## Abstract

Cancer cells often exhibit altered epigenetic signatures that can misregulate genes involved in processes such as transcription, proliferation, apoptosis and DNA repair. As regulation of chromatin structure is crucial for DNA repair processes, and both DNA repair and epigenetic controls are deregulated in many cancers, we speculated that simultaneously targeting both might provide new opportunities for cancer therapy. Here, we describe a focused screen that profiled small-molecule inhibitors targeting epigenetic regulators in combination with DNA double-strand break (DSB) inducing agents. We identify UNC0638, a catalytic inhibitor of histone lysine N-methyl-transferase G9a, as hypersensitising tumour cells to low doses of DSB-inducing agents without affecting the growth of the non-tumorigenic cells tested. Similar effects are also observed with another, structurally distinct, G9a inhibitor A-366. We also show that small-molecule inhibition of G9a or siRNA-mediated G9a depletion induces tumour cell death under low DNA damage conditions by impairing DSB repair in a p53 independent manner. Furthermore, we establish that G9a promotes DNA non-homologous end-joining in response to DSB-inducing genotoxic stress. This study thus highlights the potential for using G9a inhibitors as anti-cancer therapeutic agents in combination with DSB-inducing chemotherapeutic drugs such as etoposide.

## Highlights

• Cancer cell growth inhibition screen with epigenetic chemical probes and phleomycin.
• G9a inhibitor UNC0638 hypersensitises tumour cells to DNA-damaging agents.
• Under low-level damage, G9a inhibitor induces p53-independent tumour cell death.
• G9a depletion induces tumour cell death by impairing DNA double-strand repair.
• G9a promotes DSB repair by non-homologous end joining.

## Introduction

Cancer cells frequently exhibit sustained proliferation, evasion of checkpoints, resistance to cell death signals, and activation of pathways that promote cancer invasion and metastasis [1]. These pathways are under intense investigation to better understand cancer biology and develop targeted therapies. Recently, it has become clear that cancer cells also often display altered epigenetic signatures at the chromatin and transcriptional levels, thus promoting research and drug discovery into epigenetic-focused cancer therapies [2].

Genes encoding enzymes that create histone methylation marks such as nuclear receptor binding SET-domain proteins 1, 2 and 3 (NSD1, NSD2 and NSD3), SET domain containing 2 (SETD2) and others undergo recurrent translocations and/or coding mutations in various cancer types (reviewed in Reference 2). Another enzyme, DOT1L, which generates the histone H3K79me2 mark, has emerged as a promising epigenetic drug target, as its small molecule-mediated inhibition selectively kills mixed lineage leukaemia (MLL) cells [3]. Heterodimeric methyl-transferases G9a/EHMT2 and GLP/EHMT1 mediate monomethylation and dimethylation of lysine 9 of histone H3 [4]. Elevated expression of G9a is associated with poor prognosis in aggressive lung cancer, and dysregulates epigenetic pathways in lung tumorigenesis [5]. Such studies thus highlight the potential for G9a/GLP inhibitors in cancer drug development. Currently, perhaps the most prominent compounds targeting epigenetic mechanisms are those targeting bromodomain and extra-terminal motif (BET) proteins that exhibit strong efficacy in selectively killing cells comprising a wide range of haematologic malignancies [6], [7], [8], [9]. Such findings have underscored the potential of epigenetic inhibitors in treating cancer, and have received widespread attention by drug discovery companies, leading to development of small-molecule inhibitors that specifically target epigenetic enzymes and chromatin readers/regulators.

To allow the scientific community to have open access to newly generated chemical probes targeting various proteins, including those that mediate epigenetic control mechanisms, the Structural Genomics Consortium (SGC) has established a partnership with more than ten pharmaceutical companies [10]. With a mandate to create well-characterised inhibitors (chemical probes) for epigenetic targets, SGC, in collaboration with pharmaceutical companies, has developed multiple selective and potent small-molecule inhibitors targeting epigenetic enzymes and chromatin regulators. All SGC chemical probes are potent (<100 nM measured in biochemical or biophysical assays), show target selectivity and display cellular activities [10]. These properties make SGC chemical probes suitable reagents to understand the biology of the cellular pathways that they inhibit. Furthermore, they provide opportunities for exploring the potential for such inhibitors as cancer therapeutics, either alone or in combination with other agents.

Genotoxic drugs that either intercalate into double-stranded DNA or induce DNA breaks by blocking enzymes at the replication fork are widely used as chemotherapeutics for cancer treatment. For example, topoisomerase-targeting anti-cancer drugs such as etoposide and doxorubicin are commonly used for treating various cancer types. Nevertheless, while these drugs are effective in killing rapidly dividing cancer cells due to their genotoxic properties, they are also cytotoxic to normal dividing cells and are thus associated with bone marrow suppression and cardiotoxicity [11]. Furthermore, etoposide has been suggested to trap topoisomerase II β (Top2β) in addition to Top2α, which could lead to secondary leukaemia in patients [12]. In light of these issues, alternative or combinatorial approaches that might reduce the dosage of these drugs might provide new opportunities for safer and more effective use of such chemotherapeutic agents.

Epigenetic regulators function in the context of chromatin and, besides controlling gene expression, also play key roles in DNA replication, DNA repair and DNA-damage signalling [13]. For example, dynamic regulation of acetylation events on histone lysines H3K56 and H4K16 has been implicated in such responses, as well as recruitment of histone deacetylases HDAC1 and HDAC2 to sites of DNA double-strand breaks (DSBs) [14]. Inhibition of these HDACs impairs the non-homologous end joining (NHEJ) pathway of DSB repair, thus highlighting the importance of acetylation and deacetylation events at sites of DNA damage. More recent studies have established that repair-pathway choice for DSBs is also strongly influenced by epigenetic marks [15]. For instance, SETD2 mediated trimethylation of histone H3K36 on actively transcribing regions directs the homologous recombination (HR) machinery to repair DNA breaks in these regions, while DSBs in intergenic or not actively transcribed regions are repaired mainly by NHEJ [15]. These studies highlight how inhibiting epigenetic pathways might also deregulate certain DNA repair pathways. Considering that both epigenetic and DNA repair pathways are lost and/or aberrantly regulated in cancers, we hypothesised that targeting both epigenetic and DNA repair pathways simultaneously might strongly impede cancer cell proliferation and provide new opportunities for cancer therapy.

To investigate the potential of small-molecule inhibitors targeting epigenetic regulators for cancer treatment, we performed a focused screen by treating human osteosarcoma cancer cells with a library of epigenetic inhibitors obtained from SGC, in combination with the DNA damaging agents phleomycin and etoposide. This identified the G9a inhibitor UNC0638 as hypersensitising osteosarcoma cancer cells to phleomycin and etoposide without marked effects on normal epithelial cells or fibroblasts. Furthermore, we established that, under low DNA damage conditions, G9a inhibition impedes cell growth independent of p53 status, and that G9a promotes NHEJ. We discuss the potential for using G9a inhibitors in combination with DNA damaging agents, such as etoposide, for treating cancers either proficient or defective in p53.

## Materials and methods

### Cell lines

Cell lines were cultured at 37 °C under 5% CO_2_ humidified atmosphere. U2OS, HCT116 (p53 WT and KO), MRC5 cell lines were cultured in DMEM (Sigma-Aldrich) containing 10% fetal bovine serum (FBS, Gibco), 100 U/ml penicillin (Gibco), 100 µg/ml streptomycin (Gibco), and 292 µg/ml L-glutamine (Gibco). RPE1 cells were cultured in DMEM nutrient-mixture F12-HAM (Sigma-Aldrich) containing 0.23% of sodium bicarbonate (Sigma-Aldrich) and the above-mentioned additives.

### siRNA transfections

siRNA oligonucleotides were purchased from MWG Biotech. Transfections were performed using Lipofectamine RNAiMAX (Life Technologies) at ~40 nM final concentration for 72 h. siRNA targeting Luciferase (siContl) was used as negative control. Supplementary Table S2 provides a complete list of siRNA sequences.

### IncuCyte cell proliferation assays

For screening/other sensitivity experiments, cells were seeded at ~10% confluence in 96-well plates and treated with compounds 24 h afterwards. After 1 h of compound treatment, cells were treated with phleomycin (Melford Labs Ltd)/etoposide (Sigma-Aldrich) or carboplatin (Sigma-Aldrich) and cultured in an incubator with an IncuCyte microscopy-platform (Essen BioScience). For siRNA-transfected cell experiments, cells were seeded onto 96-well plates 48 h post-transfection with a similar compound/drug treatment regime. Phase-contrast images were acquired every 6 h for 6–10 days until vehicle-treated cells reached 100% confluence. Cell proliferation was monitored as the occupied area (% confluence) of cell images over time and calculated either as percentage confluence over time or percentage growth rate (change in % confluence over a period of time).

### Apoptosis assays

Cells were cultured for 3 days with different treatments and split on the third day adding fresh compound/drug combination. After 24 h, cells were washed with PBS and detached with PBS/2.5 mM EDTA. Annexin V and propidium iodide (PI) staining was performed as per the protocol provided in the Annexin V-FITC apoptosis detection kit (ab14085). Staining was quantified by flow cytometry analysing Annexin V-FITC binding with fluorescein isothiocyanate (FITC) and propidium iodide (PI) staining by the phycoerythrin emission signal detectors.

### Immunofluorescence studies

U2OS cells were treated with different treatments for 3 days, reseeded on poly-lysine coated coverslips and cultured with fresh compound/drugs for another day. Cells were washed with PBS, fixed in 4% paraformaldehyde, permeabilised with PBS/0.2% Triton X-100 and blocked with 5% BSA/PBS containing 1% Tween-20. Immunostaining was done with the indicated primary antibodies and appropriate secondary antibodies both diluted in blocking buffer. Antibody details are provided in Supplementary Table S2. Images were taken with an SP5 confocal microscope (Leica) and quantified using Volocity® 6.2.1 image analysis software (PerkinElmer).

### Immunoblotting

Cell extracts were prepared with CSK buffer (10 mM PIPES pH 6.8, 3 mM MgCl_2_, 1 mM EGTA, 0.1% Triton X-100, 300 mM sucrose and 300 mM NaCl) containing 1× protease-inhibitor cocktail (Roche), 1× phosphatase-inhibitor cocktail (Sigma-Aldrich), 10 mM N-ethylmaleimide (Sigma-Aldrich) and 0.25 mM phenylmethylsulphonyl fluoride (PMSF, Sigma-Aldrich). Cells were washed twice with ice-cold PBS, incubated with CSK buffer for 30 min on ice with occasional mixing, scraped and sonicated. Equal concentrations of protein samples were prepared, boiled, and resolved on 4–12% Bis–Tris gradient gels (Invitrogen). Separated proteins were transferred to nitrocellulose membranes (GE Healthcare) and probed with indicated antibodies.

### Neutral comet assays

U2OS cells were treated with G9a inhibitor (UNC0638, 1 µM) or ATM inhibitor (KU55933 [16], 10 µM) for 24 h, while HCT116 p53 WT/KO cells were treated for 4 days. For siRNA-transfected cells, assays were performed 72 h after transfection. Neutral comet assay were performed with the damaging agent phleomycin, as previously reported [17]. Briefly, an appropriate number of cells were mixed with low-melting Agarose (Trevigen) and bound on GelBond film (Lonza). Samples were lysed and electrophoresed at 35 V for 7 min. The samples were fixed, dried and stained with SYBR Green I (Invitrogen). Images were taken with an IX71 fluorescent microscope (Olympus) using Cell^F software (Olympus). Tail moments were quantified using CometScore software (TriTek). Means of tail moments of at least 50 cells were measured per condition.

### Random plasmid integration assay

Assays were performed as previously described [18]. Briefly, 2 days after siRNA transfection, U2OS cells were transfected with BamHI–XhoI-linearised pEGFP-C1 (Clontech). Cells were plated on two 15-cm plates, one for measuring seeding density (non-selected media) and another for random integration of the plasmid (selected with 0.5 mg/ml G418), and cultured for 10–14 days for colony formation. Transfection efficiency (GFP) was assessed by flow cytometry. Colonies were stained with 0.5% crystal violet/20% ethanol and counted. Random plasmid integration events were normalised to transfection and plating efficiencies.

### EdU click reaction for cell cycle proliferation assay

U2OS cells were pulse-labelled with 10 µM of the nucleotide analogue 5-ethynyl-2′-deoxyuridine (EdU). EdU-incorporated cells were fixed using 4% paraformaldehyde, washed with PBS, permeabilised with 0.2% Triton X-100 for 30 min, PBS washed and treated with click-reaction mixture for 30 min in the dark. Cells were washed twice in PBS containing 0.2% Triton-X and resuspended in PBS containing the nuclear stain 4′,6-diamidino-2-phenylindole dihydrochloride (DAPI). Detection of the newly incorporated nucleotide analogue was achieved via reaction of the ethynyl group with a small fluorescent azide-containing probe (Click-iT® EdU Alexa Fluor® 647 Imaging Kit no. C10340; Invitrogen). Percentages of cells in different cell cycle stages were measured by flow cytometry using a BD LSRFortessa cell analyser (BD Biosciences).

### Homologous recombination assay

Homologous recombination (HR) efficiency under control conditions, or upon G9a inhibition or depletion was monitored using the Traffic Light Reporter (TLR) system [19]. The assay was performed as described previously [20]. The TLR reporter system, stably integrated in U2OS cells, comprises a mutant GFP (green-fluorescent protein) gene with a unique recognition site for the endonuclease I-SceI. Upon transient expression of I-SceI, the repair of the induced DSB can generate a functional GFP gene when HR occurs through use of sequences in a co-transfected donor plasmid, which contains a truncated GFP functional for the part mutated in the reporter system. Therefore, GFP expression was used as a read-out for HR efficiency. U2OS TLR cells were treated with the indicated siRNA or inhibitor, and 6 h later were co-transfected with infrared fluorescent protein (IFP)-I-SceI endonuclease and blue fluorescent protein (BFP)-donor plasmid. Cells were harvested 72 h after siRNA transfection. Percentages of GFP+ (HR) cells were measured by flow cytometry using a BD LSRFortessa cell analyser (BD Biosciences). Approximately 10,000 doubly transfected (IFP+ and BFP+) cells were scored for each condition in three independent experiments. Results were normalised to Control siRNA or vehicle treated cells.

### Statistical and quantitative analysis

Statistical analyses were done by unpaired Student's t-test. For quantitative analysis, all values represent means of three independent experiments ±standard deviation (SD).

## Results

To assess the impact of recently developed epigenetic probe compounds on DNA repair and related processes, we obtained a library of eleven small-molecule compounds from the SGC that inhibit the activity of a series of distinct epigenetic regulators. These compounds, which impact on histone acetylations, methylations or chromatin–protein interactions, were generated to possess a target specificity of more than 30-fold over other proteins in the same protein family and activity in cells below 1 µM. Details of the target specificity and cellular activity of the probes used in this study are available on the SGC website (http://www.thesgc.org/chemical-probes/epigenetics).

To explore the effects of these compounds on cellular responses to DNA damage, we assessed their impacts on the sensitivity of human osteosarcoma U2OS cancer cells to agents that generate DNA DSBs. First, we assayed for the cytotoxicity of probe compounds towards U2OS cells by treating them with various doses of the epigenetic probes. Thus, we used 50 µM as the highest concentration and serially diluted each inhibitor three-fold to reach 20 nM, thereby generating dose-dependent survival curves to determine each compound's IC50 value (the concentration that led to a 50% drop in cell survival). The details of the probes used, their specific targets, references and IC50 values calculated for U2OS cells are provided in Supplementary Table S1. Based on the calculated IC50 values and data provided by SGC for each of the probes in other cell lines (available on the SGC website), we used 1 µM as the concentration for each compound for subsequent drug-combination screening. For combination studies, we chose concentrations of DNA-damaging agent phleomycin that were not overly toxic to the U2OS cells but did produce cytotoxicity when combined with inhibitors of the DDR kinases ATM or ATR (data not shown). We thus chose 0.5 µM and 1 µM of phleomycin as concentrations for the drug-combination screen.

**Fig. 1:**
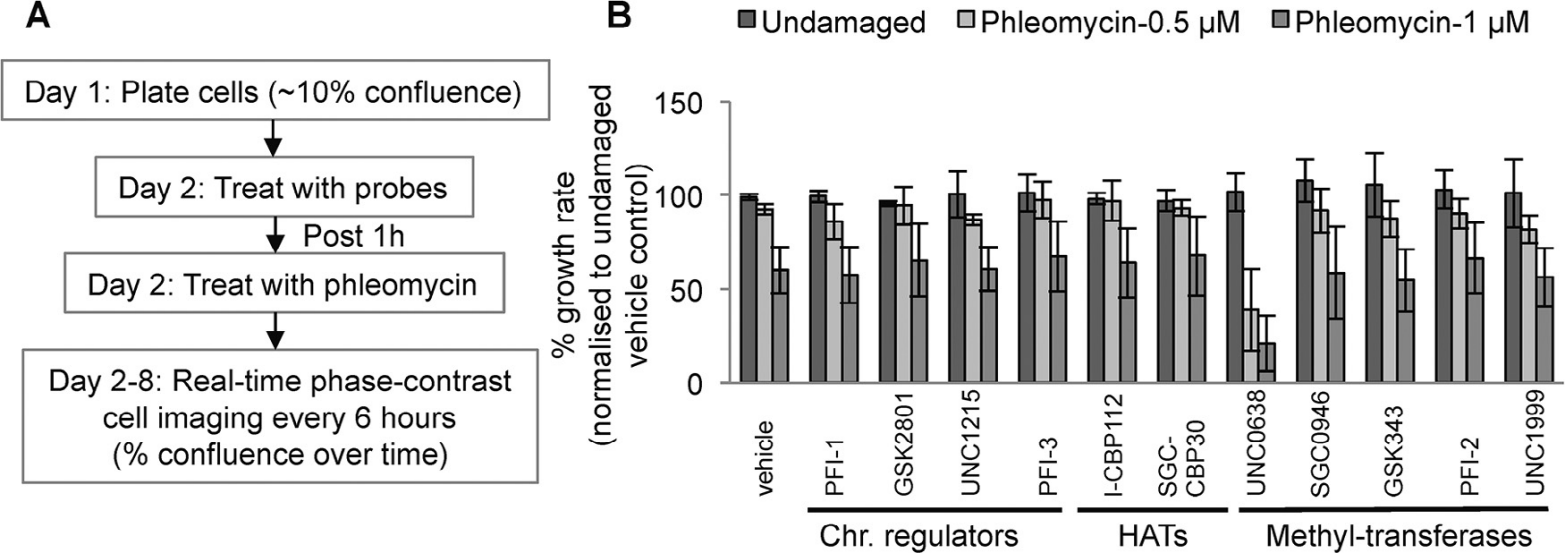
Screening chemical probe inhibitors identifies UNC0638 as potentiating with phleomycin to impede cell proliferation. (A) Schematic elaborating the screening strategy. (B) U2OS cells were seeded in 96 well plates at ~10% confluence and treated with vehicle (1% DMSO), small-molecule inhibitors targeting chromatin modulators (Chr. Regulators; PFI-1, GSK2801, UNC1215, PFI-3), histone acetyl-transferases (HATs; I-CBP112, SGC-CBP30) and histone methyl-transferases (UNC0638, SGC0946, GSK343, PFI-2, UNC1999). Details of inhibitors used are provided in Supplementary Table S1. One-hour post treatment, cells were damaged with low doses of phleomycin (0.5 µM or 1 µM) and were allowed to proliferate for 6 days. The area occupied by cells (% confluence) over time is a surrogate marker for proliferation. Data presented here provide percentage growth rates of cells over a period of 6 days. Error bars correspond to SDs of three independent experiments. Small-molecule Inhibitor UNC0638 inhibiting G9a methyl-transferase hypersensitised U2OS cells to DSB-inducing agent phleomycin at both phleomycin concentrations tested.

Next, we carried out combination drug screening by plating U2OS cells to ~10% confluence, then 24 hours later adding probe compounds for 1 hour before adding phleomycin (Fig. 1A). Cells were then assessed for growth by phase-contrast imaging every 6 hours for several days. Fig. 1B depicts ensuing data for epigenetic probe inhibitors targeting chromatin modulators (PFI-1, GSK2801, UNC1215, PFI-3), histone acetyl-transferases (I-CBP112, SGC-CBP30) and histone methyl-transferases (UNC0638, SGC0946, GSK343, PFI-2, UNC1999). Strikingly, while most probes had little or no effect on cell growth in these studies, UNC0638 markedly hypersensitised U2OS cells to phleomycin in a dose-dependent manner (Fig. 1B). UNC0638 is a substrate-competitive inhibitor that inhibits the catalytic activity of the G9a/GLP histone methyl-transferase, which has been found to mainly generate dimethylation of lysine residue 9 of histone H3 (H3K9me2) [21]. The structure of UNC0638 and a co-crystal structure of the inhibitor bound to the G9a catalytic domain are provided in Supplementary Fig. S1. While it remains to be determined whether the other probe compounds we tested have effects in combination with other DNA-damaging drugs, we selected UNC0638 for our further analyses, as described below.

**Fig. 2:**
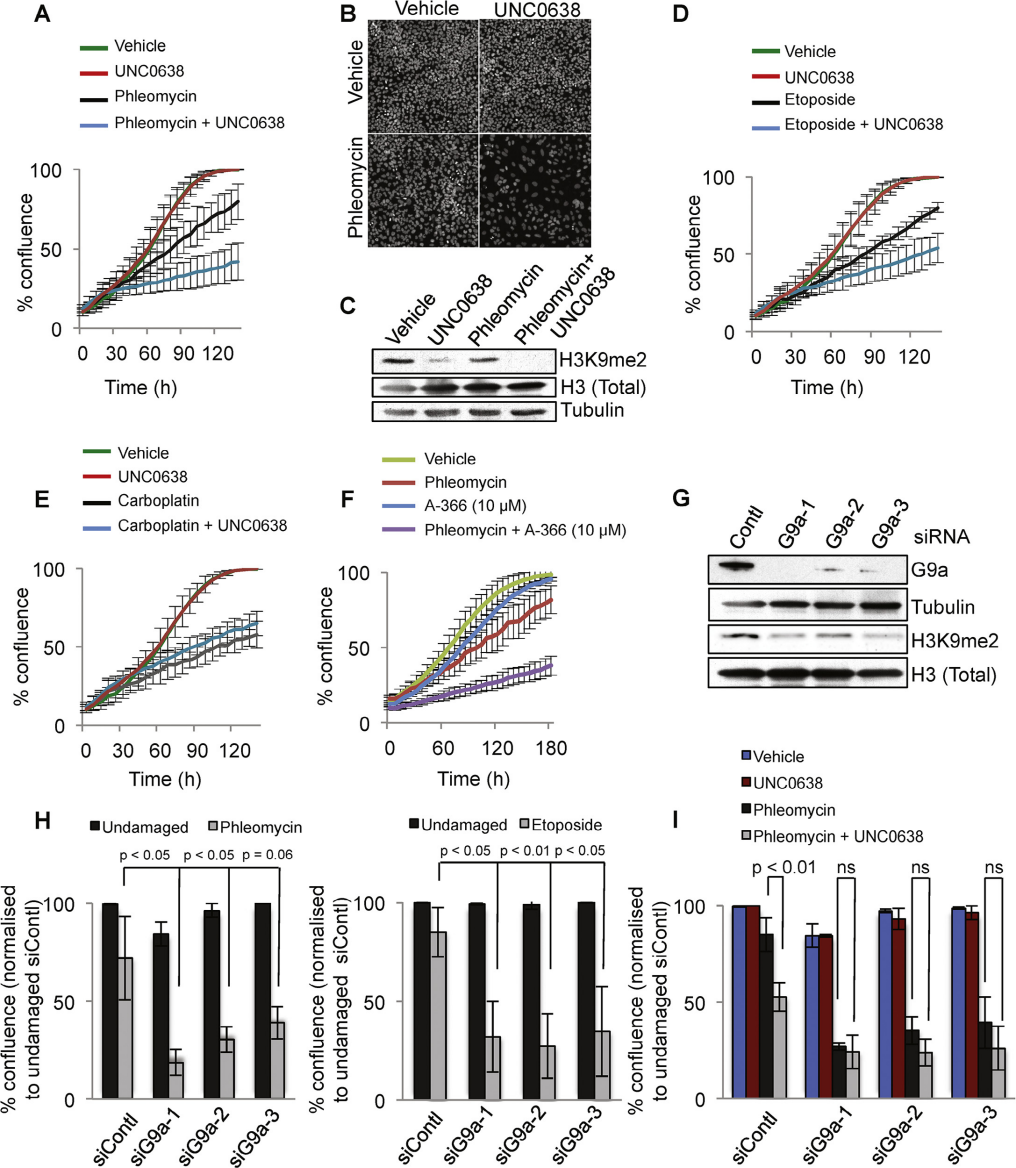
G9a inhibition potentiates the anti-tumour activity of DSB-inducing agents. (A) Growth curves for U2OS cells show that co-treatment of UNC0638 (1 µM) with phleomycin (1 µM) significantly inhibits tumour cell growth compared to cells treated with phleomycin alone. (B) Representative images of DAPI stained cell nuclei at the end-point of the proliferation assay are shown. (C) Western blot analysis reveals a reduction in levels of histone H3K9 dimethylation (H3K9me2) upon UNC0638 treatment. Total levels of histone H3 and tubulin served as loading controls. (D and E) Growth curves of U2OS cells demonstrate that co-treatment of UNC0638 (1 µM) hypersensitised tumour cells to etoposide (100 nM) but not carboplatin (15 µM). At the same concentrations of phleomycin and etoposide, UNC0638 did not adversely affect the cell growth of non-tumorigenic RPE1 and MRC5 cells (see Supplementary Fig. S3). (F) Structurally distinct chemotype of G9a small-molecule inhibitor, A-366, similarly hypersensitises U2OS cells to phleomycin. (G) Western blots confirm G9a depletion and corresponding reductions in H3K9 dimethylation (H3K9me2) upon treatment of U2OS cells with three independent siRNAs (siG9a-1, 2, 3) compared to control siRNA-transfected cells (siContl). Tubulin and total histone H3 were used as loading controls. (H) Depletion of G9a, the target of UNC0638 and A-366, with three independent siRNAs (siG9a-1, 2, 3) hypersensitises U2OS cells to phleomycin (1 µM) and etoposide (100 nM) compared to control siRNA-transfected cells (siContl). Histograms depict % confluence of cells at the endpoint of the proliferation assays. (I) Histograms provide the % confluence of cells at the endpoint of the proliferation assays with indicated treatments of U2OS cells showing that UNC0638 does not further increase the hypersensitivity of G9a depleted cells (siG9a-1, 2, 3) to phleomycin (1 µM) supporting the effects of UNC0638 being via G9a inhibition. Error bars correspond to standard deviations (SDs) of three independent experiments. Non-significant p-values are represented as ns.

As indicated by growth curves (Fig. 2A), UNC0638 treatment markedly inhibited the growth of U2OS cells in the presence of low levels of phleomycin but had little or no effect on cell growth in the absence of phleomycin. At the end-point of this experiment, plates were stained with DAPI to monitor the total number of cells after different treatments, thereby demonstrating that cell growth inhibition was also associated with inhibition of proliferation as evidenced by there being considerably reduced numbers of cell nuclei (Fig. 2B). The functionality of G9a inhibitor UNC0638 in this study was established by us observing that it reduced cellular levels of dimethylation of histone H3K9 as measured by western immunoblotting (Fig. 2C).

Next, we investigated whether the effect for UNC0638 in combination with phleomycin also extended to combinations with other clinical DNA-damaging agents. Thus, we performed cell growth assays in a similar manner but with etoposide, a Topoisomerase II inhibitor that generates enzyme-bound DSBs, or with carboplatin that induces inter- and intra-strand DNA crosslinks. Notably, UNC0638 affected cell growth when combined with etoposide (Fig. 2D) but not with carboplatin (Fig. 2E) suggesting that G9a plays a specific role in DNA DSB repair. While UNC0638 enhanced the induction of markers of DNA damage caused by treating cells with phleomycin, it had little or no effect on such markers when combined with carboplatin (Supplementary Fig. S2A and B). Interestingly, this growth inhibitory potentiating effect of UNC0638 with DSB-inducing agents showed specificity for U2OS cancer cells, as this effect was absent or not as strong in non-tumorigenic RPE-1 (retinal pigment epithelial) cells or MRC5 cells derived from lung fibroblasts (Supplementary Fig. S3). These observations thus highlighted the potential for G9a inhibition by UNC0638 and derivatives to specifically inhibit tumour cell growth in combination with low levels of DSB-inducing agents.

To gain some assurance that the responses we observed were not due to an off-target effect of UNC0638, we used another small-molecule G9a inhibitor of a different structural chemotype, A-366 [22]. Importantly, A-366 produced similar potentiating effects on U2OS cell growth and damage accumulation, when combined with phleomycin as we observed with UNC0638 (Fig. 2F, Supplementary Fig. S2C). However, while UNC0638 had effects at 1 µM, A-366 only displayed activity when used at the higher concentration of 10 µM. Because of its greater potency in these assays in combination with phleomycin, we focused on the G9a inhibitor UNC0638 in our ensuing studies. To further validate our conclusion that the potentiating effects of UNC0638 and A-366 were being mediated by them targeting G9a, we depleted G9a in U2OS cells by using three independent short-interfering RNAs (siRNAs) and evaluated the sensitivities of control and depleted cells to phleomycin or etoposide. Western immunoblot analyses established that the three siRNAs produced comparable efficiencies of G9a depletion and concurrent reduction in levels of H3K9me2 (Fig. 2G). Importantly, while low levels of phleomycin or etoposide did not appreciably affect the growth of U2OS cells transfected with control siRNA (siContl), each of the three siRNAs targeting G9a (siG9a -1, 2, 3) resulted in significant sensitisation to both phleomycin and etoposide (Fig. 2H). Furthermore, unlike the situation in control cells, where UNC0638 caused sensitisation to phleomycin, UNC0638 had little impact in further sensitising cells that had been pre-treated with siRNAs to deplete G9a. This “epistatic” relationship between G9a depletion and UNC0638 treatment thus further established the on-target effect of small-molecule mediated G9a inhibition (Fig. 2I).

**Fig. 3:**
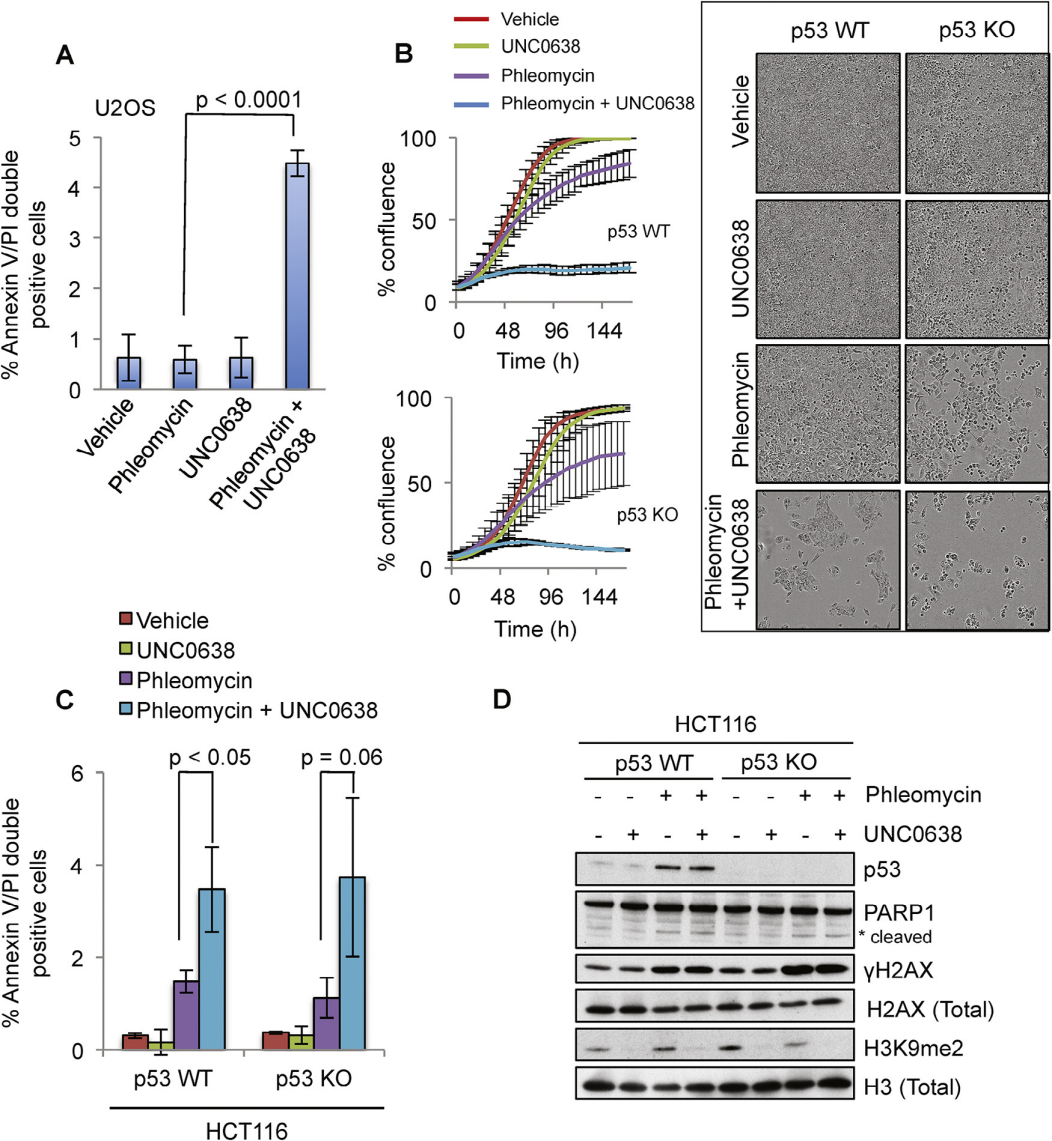
Under low damage condition UNC0638 induces tumour cell death independent of p53 status. (A) U2OS cells were treated as indicated for 4 days and stained with Annexin V and PI for flow cytometry analyses. Combined treatment of UNC0638 (1 µM) and low dose phleomycin (1 µM) significantly increases U2OS cell death as measured by percentage of Annexin V/propidium iodide double-positive cells compared to cells treated with phleomycin alone. Error bars correspond to SDs of three independent experiments. (B) Growth curves for HCT116 p53+/+ (WT) and p53−/− (KO) cells with indicated treatments show that co-treatment of UNC0638 (1 µM) with phleomycin (0.5 µM) inhibits growth of HCT116 cells independent of p53 status. Representative phase-contrast images of cells at the end-point of the confluence assay are shown. (C) HCT116 p53 WT and KO cells were treated with indicated treatments for 4 days and stained with Annexin V and PI for flow cytometry analyses. UNC0638 treatment in the presence of low dose phleomycin increases the cell death (% Annexin V/propidium iodide double-positive cells) for both p53 WT and KO cells compared to phleomycin treatment alone. Statistical analyses were performed as in (A). (D) No increase in PARP1 cleavage was observed upon co-treatment of UNC0638/phleomycin compared to phleomycin treatment only. See also Fig. S4.

Next, we explored how the combinatorial treatment of UNC0638 and DSB-inducing agents impeded tumour cell growth, considering mechanisms impacting on cell-cycle arrest, senescence, apoptosis or other modes of cell death. We first assessed whether U2OS cells might undergo apoptosis when co-treated with phleomycin and UNC0638 (cells treated with vehicle, 1% DMSO, served as a control). UNC0638 is a reversible inhibitor and is stable for at least 3 days in cell culture conditions [21]. Thus, after treating U2OS cells with phleomycin and UNC0638 alone and in combination for four days, cells were doubly stained for FITC-conjugated Annexin V and propidium iodide (PI), then were analysed by flow cytometry to monitor apoptotic cells. Externalisation of Annexin V is a readout of early stages of apoptosis, while cells that lose cell membrane integrity show positive PI staining. We observed a significant increase in the percentage of Annexin V/PI doubly positive cells upon co-treatment of UNC0638 in the presence of low concentrations of phleomycin compared to cells treated with phleomycin or UNC0638 alone (Fig. 3A and Supplementary Fig. S4). However, we did not observe a significant increase in the percentage of Annexin V singly-positive cells upon co-treatment of UNC0638 and phleomycin (data not shown), which suggested that the mode of cell death was independent of early apoptotic processes that are mainly mediated by p53.

To directly test the relevance of p53 in mediating the observed cell death, we used HCT116 colorectal carcinoma cells inactivated for the *TP53* gene (p53 KO) [23] as well as HCT116 cells with wild-type p53 (p53 WT). Notably, we observed that combined treatment of UNC0638 and phleomycin impeded cell growth independent of p53 status (Fig. 3B). Furthermore, we observed similar increases in Annexin V/PI doubly-positive cells upon co-treatment with UNC0638 and phleomycin in both wild-type and *TP53* knockout cells (Fig. 3C and Supplementary Fig. S4). This suggested that the cell death induced by the combinatorial treatment was via a p53 independent mechanism, which could be necrosis or p53 independent apoptosis. This conclusion was further strengthened by our observation that combined treatment with UNC0638 and phleomycin led to no detectable increase in cleavage of poly(ADP-ribose) polymerase 1 (PARP1), which is a well established target of p53 mediated caspase-3 activity [24] compared to phleomycin treatment only (Fig. 3D). We also assessed whether combined treatment of UNC0638 with phleomycin might affect the cell cycle status of cancer cells. Indeed, combined treatment of phleomycin with G9a inhibitor induced G2 accumulation as determined by FACS analysis of cells incorporating the nucleotide analogue EdU co-stained with DAPI (Supplementary Fig. S5). Thus, these findings revealed that G9a inhibition in the presence of low levels of phleomycin induces both damage induced G2 delay and p53 independent cell death.

**Fig. 4:**
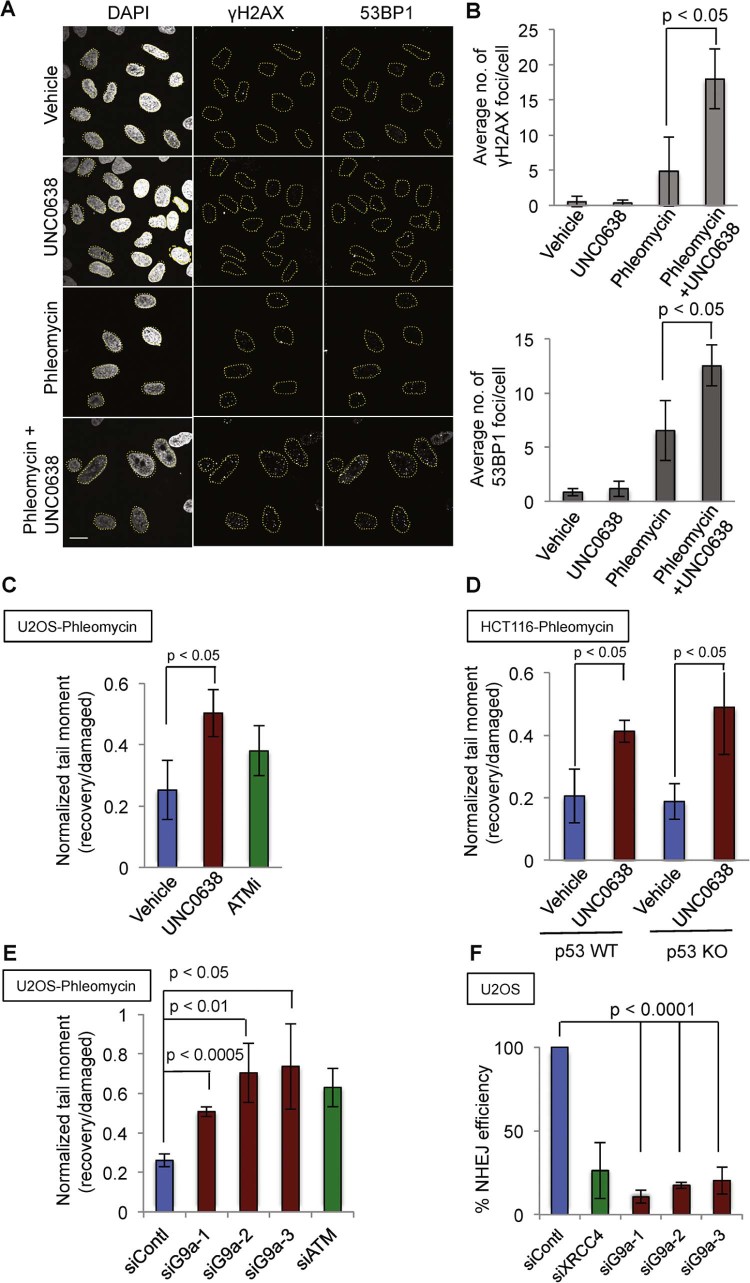
G9a inhibition impairs DNA DSB repair via NHEJ. (A) Representative immuno-fluorescent images of U2OS cells stained with antibodies recognising 53BP1, γH2AX and nuclear stain DAPI (all in grey) after indicated treatments for 4 days are shown. Dotted lines mark nuclear peripheries and the scale bar represents 10 µm. (B) Quantification of average numbers of γH2AX and 53BP1 foci per cell upon the treatments indicated in (A). Error bars correspond to SDs of three independent experiments (>100 cells were analysed per condition per experiment). Combined treatment of UNC0638 with phleomycin significantly increases the average number of γH2AX and 53BP1 foci per cell compared to phleomycin treatment alone. (C and D) DNA repair efficiencies were assayed by neutral comet assay. After the indicated treatments, U2OS and HCT116 p53 WT and KO cells were damaged with phleomycin (26 µM) for two hours (damaged), and were allowed to repair for 2 hours (recovery) after washing off the phleomycin, in the presence of indicated treatments. DSB repair efficiency was measured as the ratio of comet tail moments in recovery by damaged condition. UNC0638 treatment impaired DSB repair both in U2OS and HCT116 cells. (E) Depletion of G9a using three independent siRNAs (siG9a-1, 2, 3) impaired DSB repair upon phleomycin treatment. Depletion of ATM Kinase (siATM) served as a positive control. Comet assays were conducted as in (C). (F) Percentage efficiency of NHEJ upon depletion of G9a with three independent siRNA (siG9a-1, 2, 3) measured by random plasmid integration. Depletion of XRCC4 (siXRCC4) and control siRNA (siContl) served as positive and negative controls, respectively. Error bars correspond to SDs of three independent experiments. See also Fig. S6.

To explore the possibility that UNC0638 was inhibiting the repair of DSBs produced by phleomycin, we took advantage of the fact that unrepaired DSBs lead to the presence of subnuclear DNA-repair foci that can be visualised by staining for proteins such as 53BP1 or the DNA-damage generated, serine 139-phosphorylated derivative of histone H2A termed γH2AX [25]. Thus, we treated U2OS cells with UNC0638 and phleomycin alone or in combination for four days, and then we carried out indirect immunofluorescence staining for the DSB-markers 53BP1 and γH2AX. This revealed that cells co-treated with phleomycin and UNC0638 exhibited significantly increased numbers of γH2AX and 53BP1 foci compared to cells treated with phleomycin only (Fig. 4A and B), suggesting that they experienced higher levels of unrepaired DSBs.

Although other explanations were possible, the above data suggested that, over several rounds of DNA replication in the presence of low levels of phleomycin, DSBs were produced in U2OS cells and were resolved by a mechanism(s) that was impaired by G9a inhibition. To explore this model, we tested whether UNC0638 treatment affected DSB repair by using neutral comet assays. Thus, after cells were mock-treated or treated with phleomycin for 2 hours (damaged condition), phleomycin was removed by washing and cells were incubated for a further two hours to allow DSB repair to proceed (recovery). Comet-tail moments were then analysed in the various samples, and the ratio of comet-tail moments at recovery to damaged time-point provided a measure of DNA repair efficiency (ATM inhibitor KU55933 [16] was used as a positive control to confirm the functionality of the assay). Ensuing analyses demonstrated that treatment with G9a inhibitor UNC0638 impaired the efficiency of DSB repair in U2OS cells (Fig. 4C) as well as in HCT116 cells with or without functional p53 (Fig. 4D). Furthermore, depleting G9a using any of three independent siRNAs also led to DSB repair defects as measured by neutral comet assays (Fig. 4E; representative images showing tail moments of single cells quantified for Fig. 4C–E are depicted in Supplementary Fig. S6A–C). By western blot analyses, we also observed retention of DNA damage induced phosphorylations of ATM, KAP1 and CHK2 (p-ATM, p-KAP1 and p-CHK2) as well as γH2AX in cells treated with G9a inhibitor or G9a siRNAs compared to vehicle or control siRNA treated cells (Supplementary Fig. S6D).

In light of these data and because most DSBs in human cells are repaired by the pathway of non-homologous end joining (NHEJ), we assessed the efficiency of this pathway by a random plasmid integration assay [26]. This assay specifically determines NHEJ because it involves genomic integration of a linearised GFP-bearing plasmid with no extensive homology to the human genome. By using this assay, we found that NHEJ was substantially reduced by siRNA-mediated depletion of G9a (Fig. 4F). While NHEJ repairs DSBs in all cell cycle phases, damage in S/G2 cells is repaired by homologous recombination (HR). We tested if G9a depletion might affect the HR efficiency by quantifying integration of an HR donor plasmid by using the Traffic Light Reporter (TLR) system [19], [20] (Supplementary Fig. S7A and B). While we observed a mild HR defect upon G9a depletion, inhibiting G9a catalytic activity with UNC0638 did not significantly affect HR efficiency (Supplementary Fig. S7C; note that these assays were controlled for effects on the proportion of cells in S/G2). Collectively, these results pointed to a role of G9a in promoting DSB repair mainly by NHEJ.

## Discussion

In this study, we have established that G9a inhibition together with induction of low-level DNA damage by the DSB-inducing agents phleomycin or etoposide selectively impedes tumour cell growth without markedly affecting the growth of the normal epithelial cells or fibroblasts that we tested. Our study supports a model in which inhibition of G9a catalytic activity prevents repair of DSBs and possibly other forms of DNA damage, leading to damage persistence and ensuing reduced proliferation and death of cancer cells. We also show that this enhanced growth inhibition of cancer cells by DSB-inducing agents in the presence of UNC0638 is independent of p53 status. A previous study reported that depleting G9a using short-hairpin RNA approaches in colorectal cell lines HT29 and SW620 induced endogenous DNA damage [27]. However, in our study, we did not observe pronounced induction of DNA damage either by siRNA-mediated depletion of G9a or by using the catalytic inhibitor UNC0638 in two different cell lines: osteosarcoma U2OS and colorectal HCT116 cells. We speculate that the differences in our findings and those in the published report could be due to differences in experimental conditions or cancer cell lines used.

To develop a deeper understanding of the therapeutic potential for G9a inhibition, knowledge of tumour cell selectivity will be critical. While we observed no significant effect on growth potential of normal fibroblasts and epithelial cells upon combined treatment with G9a inhibitor/DSB agents at the doses we used, it will be important to test this and other combinations on larger collections of non-transformed and transformed cells. Nevertheless, by using isogenic p53 cell models, we found that the potentiating effects of G9a inhibition in the context of phleomycin were independent of p53 status, with tumour cells either proficient or deficient in p53 being similarly hypersensitised to phleomycin by G9a inhibition. Mutation or deficiency in p53 is associated with resistance to chemotherapy [28], and hence targets that might impede tumour cell growth independent of p53 may have considerable potential in the clinic.

We found that inhibiting G9a *per se* did not lead to detectable accumulation of DNA DSBs in cells as reflected by γH2AX and 53BP1 focus formation or by comet assays. Instead, our data indicate that it is only in the presence of a DNA-damaging agent such as phleomycin that G9a inhibition markedly affects cell proliferation, by inhibiting DSB repair. We found that the NHEJ pathway of DSB repair is markedly affected by G9a depletion, although it remains to be determined precisely how and at which step(s) G9a operates to enhance this repair pathway. As a major role for G9a is to mediate methylation of H3K9, we investigated whether impairment of DSB repair might reflect a reduction in this chromatin mark, global chromatin decompaction and concurrent transcriptional activation. However, we observed no marked reduction of H3K9 methylation within 24 hours of G9a inhibitor treatment, while this treatment was sufficient to impair DSB repair in U2OS cells. In addition, G9a inhibition/loss did not alter the protein levels of DDR proteins, such as DNA-PKcs, Ku80, XRCC4 and XLF, which are crucial for repairing DSBs by NHEJ (Supplementary Fig. S8). These findings suggest that the mechanism by which G9a inhibition/loss impairs DSB repair might be independent of global changes in chromatin compaction and associated alterations in gene expression. In this regard, we note that G9a also methylates non-histone proteins [29], some of which are already identified. It is therefore possible that G9a driven methylation of a non-histone proteins – and perhaps intrinsic components of the NHEJ apparatus – could cause this repair defect, a scenario that we are currently investigating.

In conclusion, our findings highlight the potential for using small-molecule inhibitors of G9a in combination with certain DNA-damaging chemotherapeutic agents. More broadly, our data promote the idea of testing epigenetic compounds not just as individual agents but also in combination with other chemotherapeutics. As epigenetic events participate in all processes occurring on chromatin, it will be interesting to extend studies to test synergies of epigenetic probes with agents that generate other forms of DNA damage and/or which impede DNA replication. Furthermore, there is the potential to also explore the combinatorial potential of epigenetic probe compounds with other chemotherapeutic drugs targeting a diversity of other cancer-relevant processes. In addition to providing insights into aspects of cell biology, such studies may provide additional ways to study cancer related processes, identify medical implications of combinatorial treatment regimens and provide opportunities for developing new cancer therapies.

## Conflict of interest

The authors declare no conflict of interests.
