# Supplementary material for: G9a inhibition potentiates the anti-tumour activity of DNA double-strand break inducing agents by impairing DNA repair independent of p53 status
Source: Cancer Lett. 2016 Oct 1;380(2):467–75. doi: 10.1016/j.canlet.2016.07.009 (PMC5011428; doi:10.1016/j.canlet.2016.07.009)
Supplement: Appendix S1 — Figs. S1–S8 and Tables S1–S2. [file mmc1.pdf]

Supplementary data information:

Supplemental Figures: Figures S1 – S8

Supplemental Tables: Tables I & II

Supplemental References:

Figure S1:

A

B

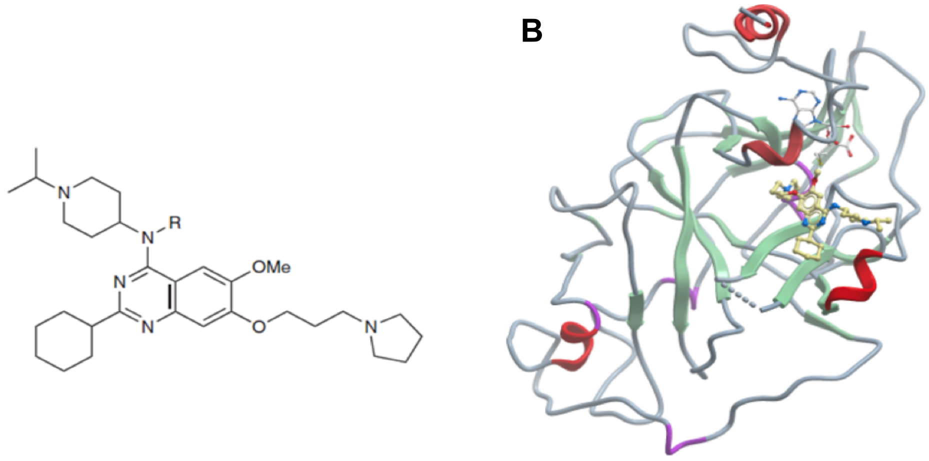

Figure S1:

(A) Chemical structure of UNC0638 [7], a small molecule inhibitor of G9a/GLP methyl-transferases (B) Co-crystal structure of the G9a–UNC0638–SAH complex [7]. The coordinates and structure factors for the co-crystal structure can be accessed from the protein databank under Pdb accession code 3RJW [7].

Figure S2:

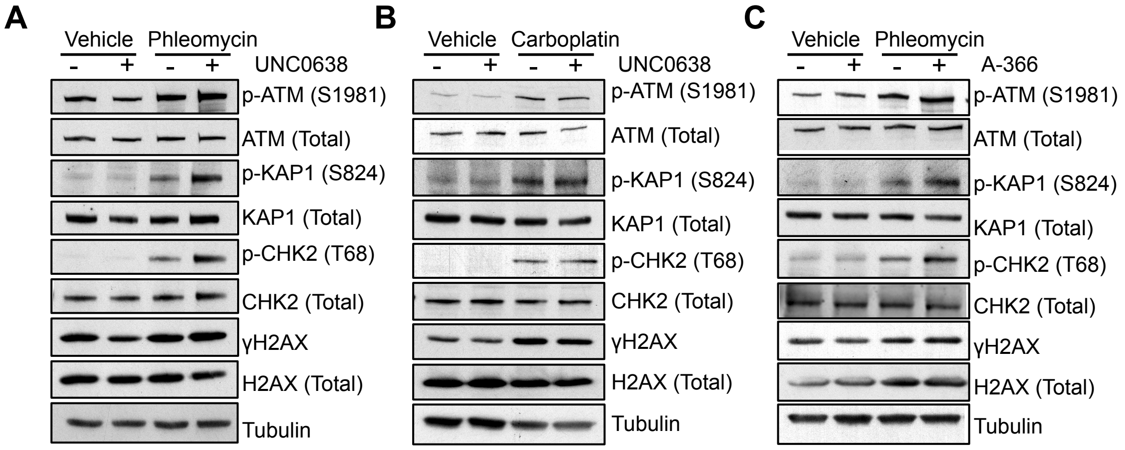

Figure S2:

(A & C) Western blots show induction of DNA damage markers (p-ATM, p-KAP1, p-CHK2 and γH2AX) upon combined treatment of UNC0638 (1 μM) and A-366 (10 μM) with phleomycin (1 μM) compared to treatment with damaging agent alone. (B) No induction was observed in cells treated with combinatorial treatment of carboplatin (15 μM) and UNC0638 compared to carboplatin treatment alone. Total protein levels of ATM, KAP1, CHK2, histone H2AX and Tubulin served as controls.

Figure S3:

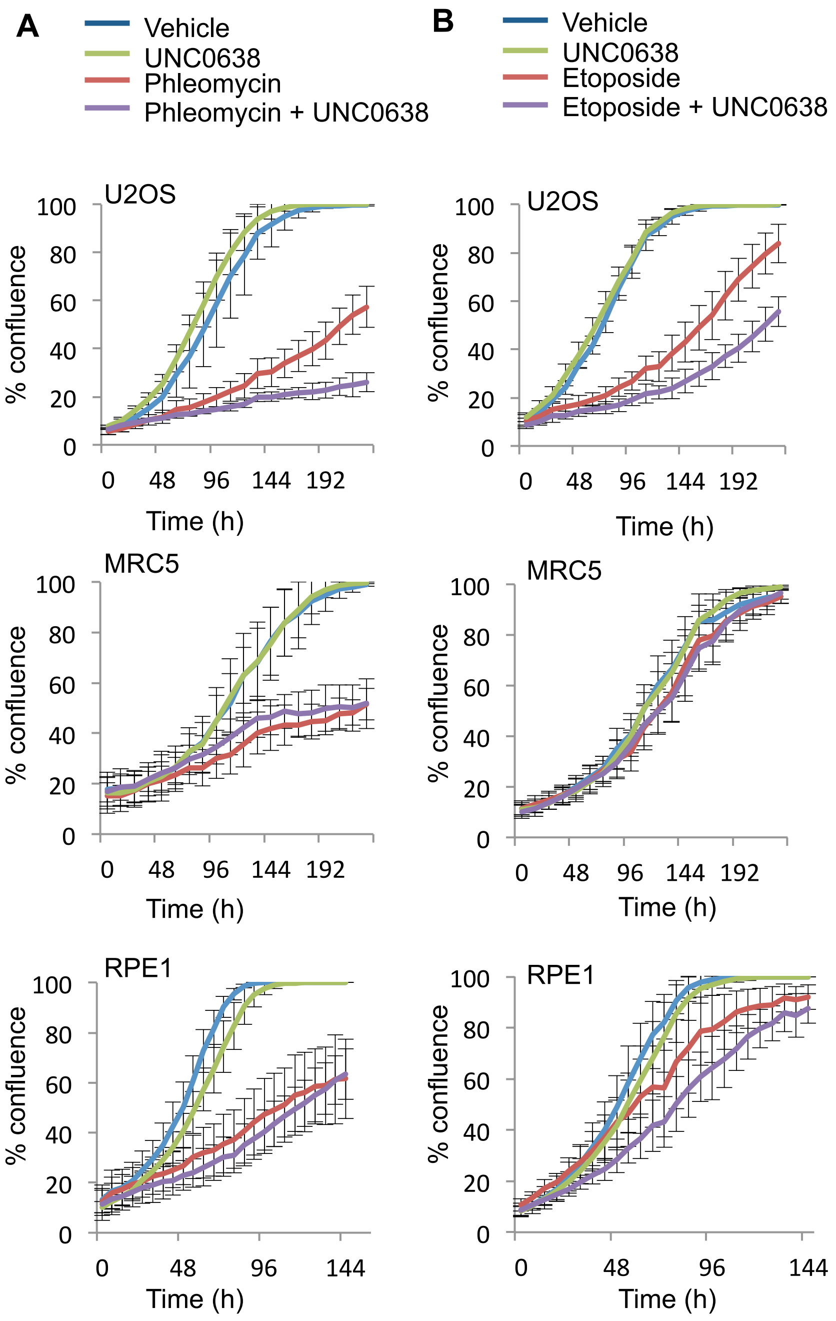

Figure S3 (related to Figure 2):

Growth curves determine hypersensitivities of U2OS, MRC5 and RPE1 cells to phleomycin (1 µM) (A) and etoposide (100 nM) (B) in combination with UNC0638 (1 µM) compared to treatments with vehicle, UNC0638 and the damaging agents, alone. UNC0638 hypersensitised cancer cells U2OS to phleomycin and etoposide, but not the non-tumorigenic RPE1 and MRC5 cells.

Figure S4:

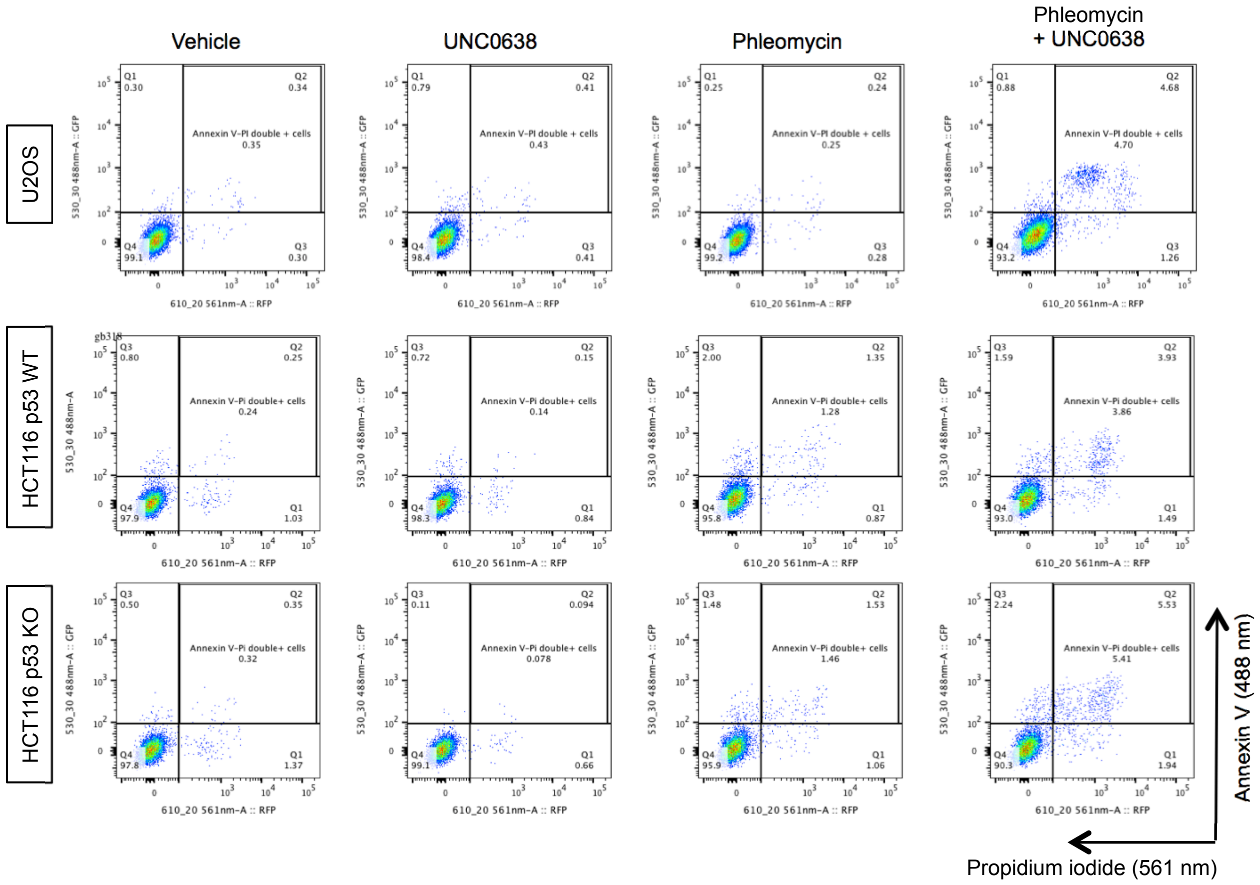

Figure S4 (related to Figure 3):

Representative FACS plots images showing Annexin V (Y-axis, 488 nm in logarithmic scale) and propidium iodide (X-axis, 561 nm in logarithmic scale) staining of U2OS and HCT116 p53+/+ (WT) and p53-/- (KO) cells under indicated treatments. Quantification of Annexin V/PI double positive cells for each condition is provided in the main figures 3A and 3C.

Figure S5:

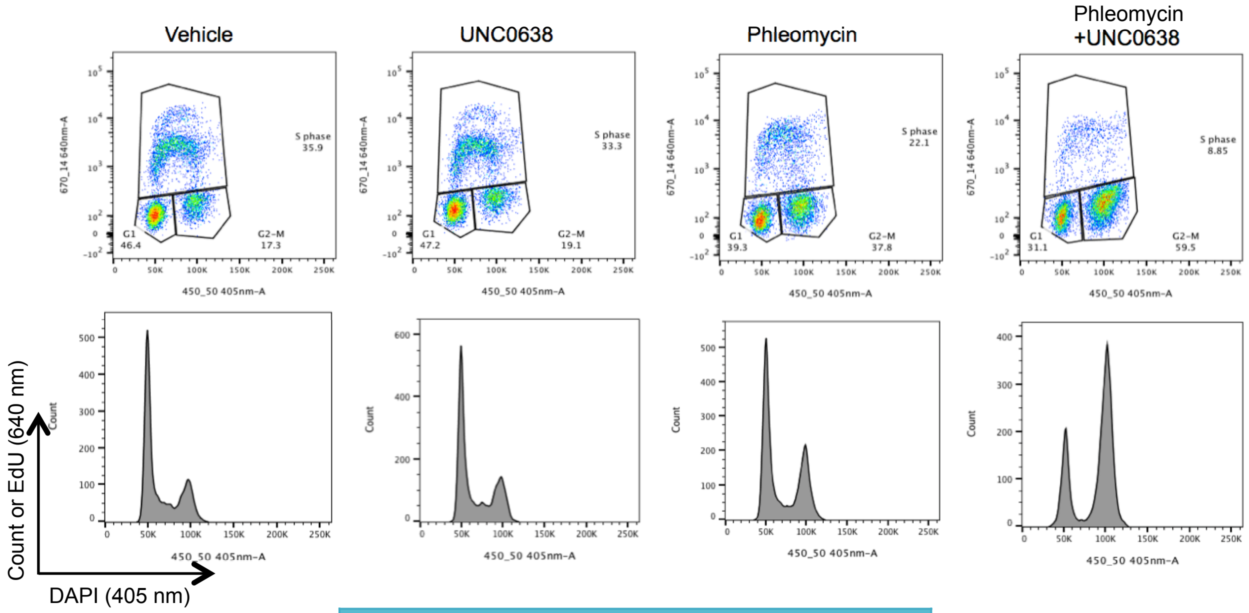

Samples	G1 (%)	S (%)	G2 (%)
Vehicle	45.9	36.6	16.4
UNC0638	44.4	36.9	18
Phleomycin	41.8	24.8	32.6
Phleomycin +UNC0638	30.1	9.2	59.3

Figure S5:

(A) U2OS cells were treated as indicated for 4 days and pulse labelled with 10 µM nucleotide analogue EdU for 30 min before fixation. Cell cycle status was monitored by FACS analyses plotting 647 nm in logarithmic scale, which measures the incorporation of the EdU (S-phase) versus the 405 nm in linear scale corresponding to DAPI content. The average numbers of cells in different phases for two independent experiments are presented in tabular form. In the presence of low levels of low damage, G9a inhibition by UNC0638 induced G2 arrest of U2OS cells.

Figure S6:

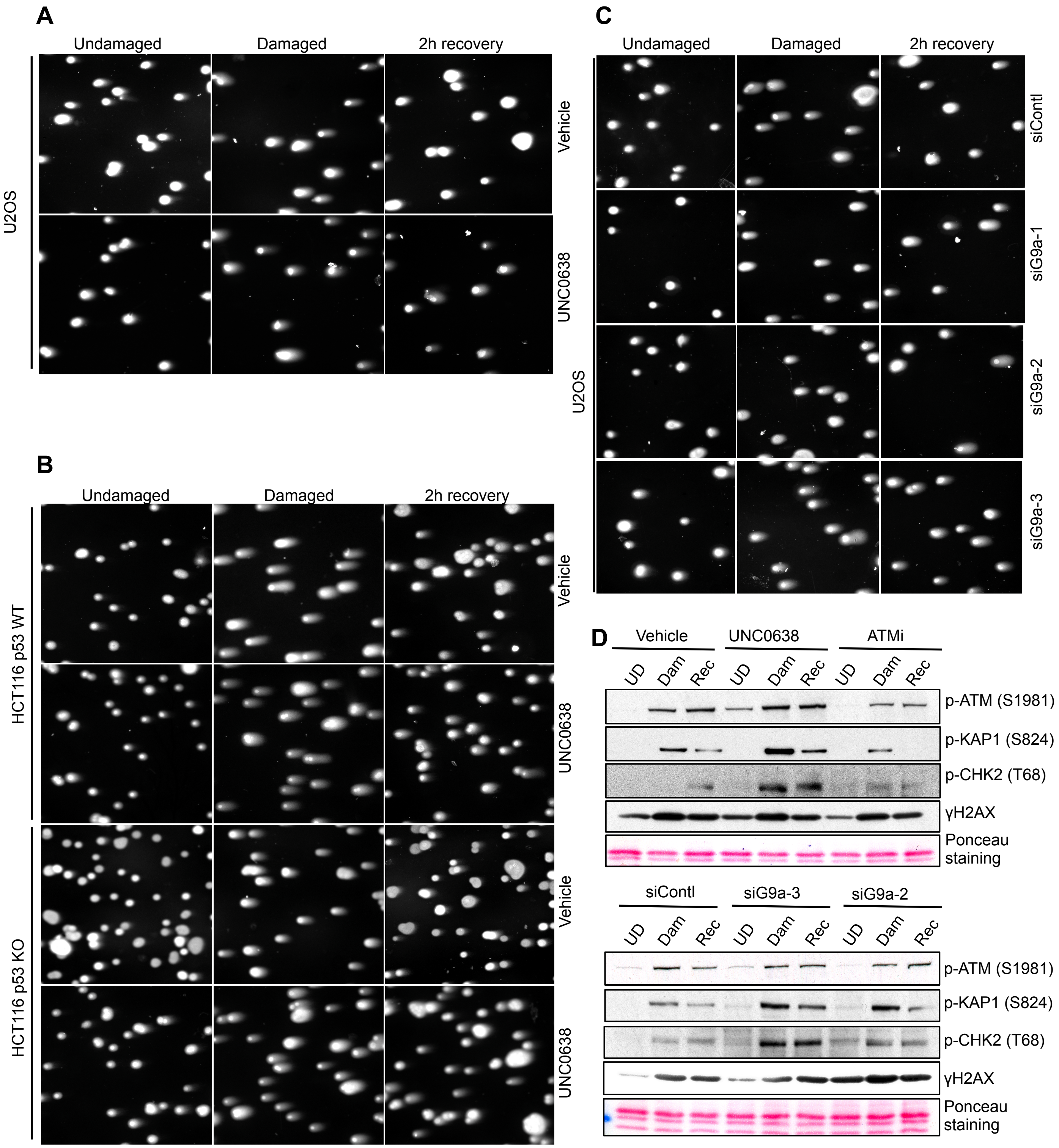
A
Undamaged
Damaged
2h recovery
U2OS
Vehicle
UNC0638
B
Undamaged
Damaged
2h recovery
HCT116 p53 WT
HCT116 p53 KO
Vehicle
UNC0638
Vehicle
UNC0638
C
Undamaged
Damaged
2h recovery
U2OS
siContl
siG9a-1
siG9a-2
siG9a-3
D
Vehicle
UNC0638
ATMi
UD
Dam
Rec
p-ATM (S1981)
p-KAP1 (S824)
p-CHK2 (T68)
γH2AX
Ponceau staining
siContl
siG9a-3
siG9a-2
UD
Dam
Rec
p-ATM (S1981)
p-KAP1 (S824)
p-CHK2 (T68)
γH2AX
Ponceau staining

Figure S6 (related to Figure 4):

(A, B & C) Representative images that correspond to neutral comet assays quantified in main figures 4C, 4D & 4E, respectively. Under damaged conditions (phleomycin treatment), the damaged DNA is visible as comet tails, which upon repair is accompanied with shrinkage of tails. UNC0638 mediated G9a inhibition or its siRNA-mediated depletion led to defective comet tail shrinkage, i.e. defective DNA repair. (D) Western blot analyses showing that, upon G9a inhibition/depletion, DNA damage induced ATM signaling was higher and generally was diminished less on recovery compared to vehicle treated/control siRNA treated cells. (UD – undamaged, Dam – phleomycin damaged, Rec – 2h recovery).

Figure S7:

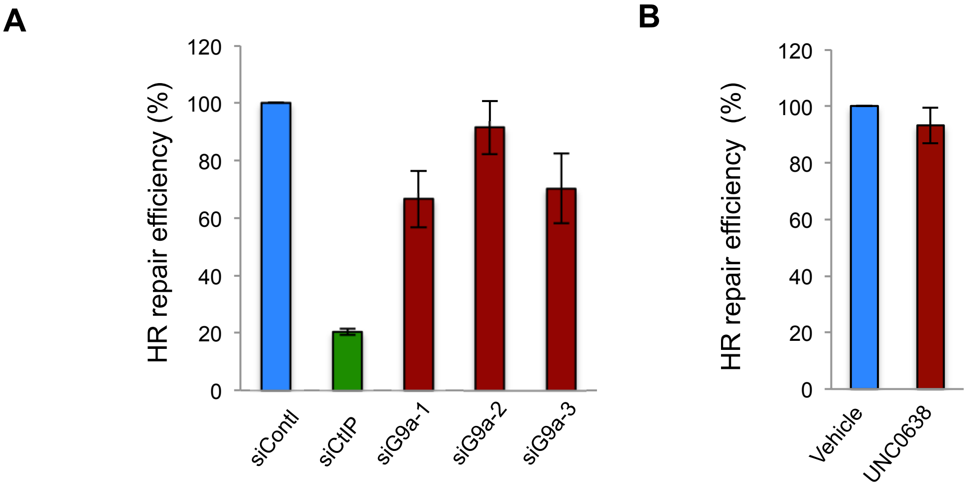

C

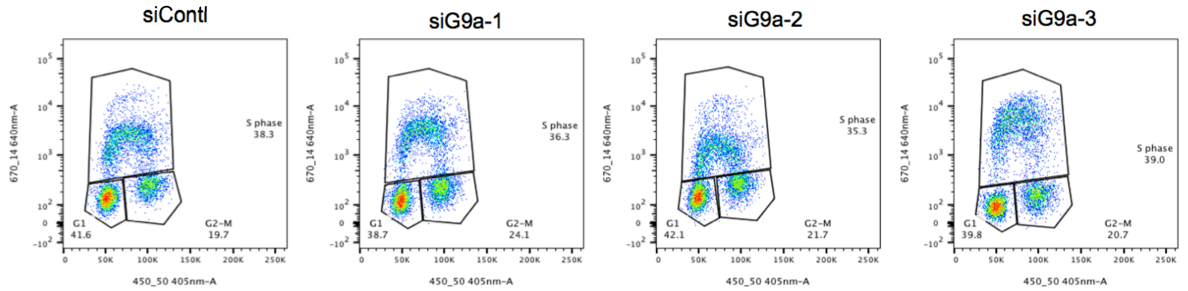

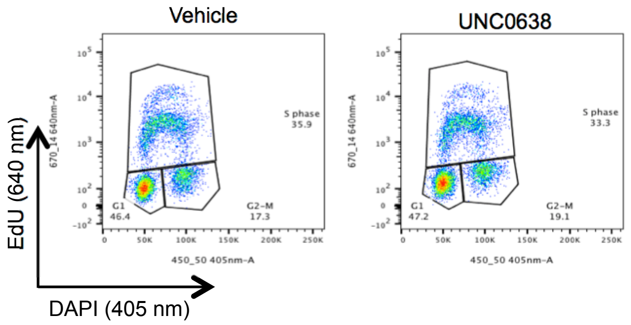

Samples	G1 (%)	S (%)	G2 (%)	S+G2 (%)
siContl	41.8	38.3	19.7	58
siG9a-si1	38.7	36.3	24.1	60.4
siG9a-si2	42.1	35.3	21.7	57
siG9a-si3	39.8	39.0	20.7	59.7
Vehicle	46.4	35.9	17.3	53.2
UNC0638	47.2	33.3	19.1	52.4

Figure S7:

(A & B) Homologous recombination (HR) repair efficiency upon depletion or catalytic inhibition of G9a as evaluated by Traffic-Light-Reporter (TLR) system in U2OS cells (refer to Materials and Methods for assay details). While G9a depletion mildly affected HR repair efficiency, no substantial effect was observed upon its catalytic inhibition. Data represent means +/- SD. (C) Cell cycle analyses confirmed no significant effect on the proportion of cells in S/G2 phase (HR occurs specifically in these cell cycle stages) upon G9a depletion/ inhibition.

Figure S8:

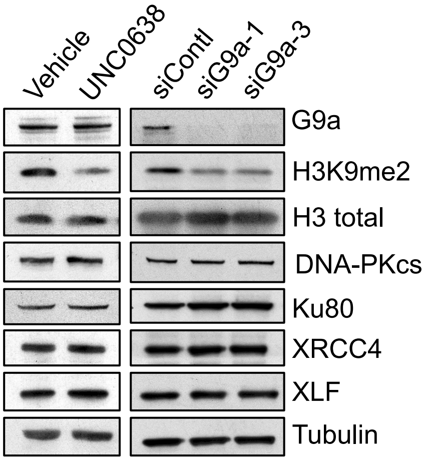

Figure S8:

Western blot analysis showing that G9a depletion or inhibition does not markedly affect the protein levels of DNA repair factors DNA-PKcs, Ku80, XRCC4 and XLF, which actively participate in the NHEJ repair pathway. Protein levels of H3K9me2, G9a, histone H3 and Tubulin levels were analysed as controls.

Supplementary Table I (related to Figure 1B):

The table provides details of the chemical probe inhibitors used in this study (refer to figure 1B) with their inhibitory concentration 50 (IC50) values for U2OS cells.

**Probe name/ References**	**Validated targets**	**Target functions**	**IC50 (μM) in U2OS**
PFI-1 [1]	BET	Chromatin regulator	0.96
GSK2801 [2]	BAZ2B/A	Chromatin regulator	6.2e11
UNC1215 [3]	L3MBTL3	Chromatin regulator	2.3e6
PFI-3 [4]	SMARCA4	Chromatin regulator	3e13
I-CBP112 [5]	CREBBP/EP300	Acetyl-transferase	23.04
SGC-CBP30 [6]	CREBBP/EP300	Acetyl-transferase	118.7
UNC0638 [7]	G9a/GLP	Methyl-transferase	3.23
SGC0946 [8]	DOT1L	Methyl-transferase	21.24
GSK343 [9]	EZH2	Methyl-transferase	12.92
PFI-2 [10]	SETD7	Methyl-transferase	173.7
UNC1999 [11]	EZH2	Methyl-transferase	9.57

Supplementary Table II:

The table provides details of siRNAs and antibodies used in this study.

List of Antibodies			
**Antibody**	**Species**	**Reference /Suppliers**	**Application**
G9a	Rabbit	Abcam (Ab133482)	IB
H3K9me2	Mouse	Abcam (Ab1220)	IB
p53	Mouse	Santa Cruz (SC-126)	IB
PARP1	Rabbit	Cell Signaling (9542)	IB
Phospho-histone H2AX (S139)	Mouse/Rabbit	Millipore (05-636)/cell signaling (2577)	IB/IF
53BP1	Mouse	[12]	IF
Tubulin	Mouse	Abcam (ab3194)	IB
Histone H3 (total)	Rabbit	Abcam (ab1791)	IB
Histone H2AX (total)	Rabbit	Abcam (ab11175)	IB
p-ATM (S1981)	Rabbit	Epitomics (2152-1)	IB
ATM (Total)	Rabbit	Abcam (ab32420)	IB
p-KAP1 (S824)	Rabbit	Bethyl Laboratories, Inc (IHC-00073)	IB
KAP1 (Total)	Rabbit	Abcam (ab10483)	IB
p-CHK2 (T68)	Rabbit	Cell Signalling Technology (2661S)	IB
CHK2 (Total)	Mouse	Millipore (05-649)	IB
DNA-PKcs	Mouse	Thermo Scientific Abgene (MS-370-P1)	IB
Ku80	Mouse	Fisher Scientific (MS-285-P1)	IB
XRCC4	Rabbit	Abcam (ab145)	IB
XLF	Rabbit	Abcam (ab33499)	IB

List of siRNAs		
**Gene**	**siRNA sequence**	**Suppliers**
Luciferase (control siRNA)	gcaugcgccuuaugaagcu	MWG Biotech
G9a (si-1)	ccaugaacaucgaucgcaa	MWG Biotech
G9a (si-2)	ucacacauuccugaccaga	MWG Biotech
G9a (si-3)	ccaacugguuccuuuuguu	MWG Biotech
ATM	gacuuuggcugucaacuuucg	MWG Biotech
XRCC4	auauguuggugaacugaga	MWG Biotech
CtIP	gcuaaaacaggaacgaauc	MWG Biotech

Supplementary References:

[1] Picaud S, Da Costa D, Thanasopoulou A, Filippakopoulos P, Fish PV, Philpott M, Fedorov O, Brennan P, Bunnage ME, Owen DR, Bradner JE, Taniere P, O'Sullivan B, Muller S, Schwaller J, Stankovic T, Knapp S: PFI-1, a highly selective protein interaction inhibitor, targeting BET Bromodomains. Cancer research 2013, 73:3336-46.

[2] Chen P, Chaikuad A, Bamborough P, Bantscheff M, Bountra C, Chung CW, Fedorov O, Grandi P, Jung D, Lesniak R, Lindon M, Muller S, Philpott M, Prinjha R, Rogers C, Selenski C, Tallant C, Werner T, Willson TM, Knapp S, Drewry DH: Discovery and Characterization of GSK2801, a Selective Chemical Probe for the Bromodomains BAZ2A and BAZ2B. Journal of medicinal chemistry 2016, 59:1410-24.

[3] James LI, Barsyte-Lovejoy D, Zhong N, Krichevsky L, Korboukh VK, Herold JM, MacNevin CJ, Norris JL, Sagum CA, Tempel W, Marcon E, Guo H, Gao C, Huang XP, Duan S, Emili A, Greenblatt JF, Kireev DB, Jin J, Janzen WP, Brown PJ, Bedford MT, Arrowsmith CH, Frye SV: Discovery of a chemical probe for the L3MBTL3 methyllysine reader domain. Nature chemical biology 2013, 9:184-91.

[4] Vangamudi B, Paul TA, Shah PK, Kost-Alimova M, Nottebaum L, Shi X, Zhan Y, Leo E, Mahadeshwar HS, Protopopov A, Futreal A, Tieu TN, Peoples M, Heffernan TP, Marszalek JR, Toniatti C, Petrocchi A, Verhelle D, Owen DR, Draetta G, Jones P, Palmer WS, Sharma S, Andersen JN: The SMARCA2/4 ATPase Domain Surpasses the Bromodomain as a Drug Target in SWI/SNF-Mutant Cancers: Insights from cDNA Rescue and PFI-3 Inhibitor Studies. Cancer research 2015, 75:3865-78.

[5] Picaud S, Fedorov O, Thanasopoulou A, Leonards K, Jones K, Meier J, Olzscha H, Monteiro O, Martin S, Philpott M, Tumber A, Filippakopoulos P, Yapp C, Wells C, Che KH, Bannister A, Robson S, Kumar U, Parr N, Lee K, Lugo D, Jeffrey P, Taylor S, Vecellio ML, Bountra C, Brennan PE, O'Mahony A, Velichko S, Muller S, Hay D, Daniels DL, Urh M, La Thangue NB, Kouzarides T, Prinjha R, Schwaller J, Knapp S: Generation of a Selective Small Molecule Inhibitor of the CBP/p300 Bromodomain for Leukemia Therapy. Cancer research 2015, 75:5106-19.

[6] Hammitzsch A, Tallant C, Fedorov O, O'Mahony A, Brennan PE, Hay DA, Martinez FO, Al-Mossawi MH, de Wit J, Vecellio M, Wells C, Wordsworth P, Muller S, Knapp S, Bowness P: CBP30, a selective CBP/p300 bromodomain inhibitor, suppresses human Th17 responses. Proceedings of the National Academy of Sciences of the United States of America 2015, 112:10768-73.

[7] Vedadi M, Barsyte-Lovejoy D, Liu F, Rival-Gervier S, Allali-Hassani A, Labrie V, Wigle TJ, Dimaggio PA, Wasney GA, Siarheyeva A, Dong A, Tempel W, Wang SC, Chen X, Chau I, Mangano TJ, Huang XP, Simpson CD, Pattenden SG, Norris JL, Kireev DB, Tripathy A, Edwards A, Roth BL, Janzen WP, Garcia BA, Petronis A, Ellis J, Brown PJ, Frye SV, Arrowsmith CH, Jin J: A chemical probe selectively inhibits G9a and GLP methyltransferase activity in cells. Nature chemical biology 2011, 7:566-74.

[8] Yu W, Chory EJ, Wernimont AK, Tempel W, Scopton A, Federation A, Marineau JJ, Qi J, Barsyte-Lovejoy D, Yi J, Marcellus R, Iacob RE, Engen JR, Griffin C, Aman A, Wienholds E, Li F, Pineda J, Estiu G, Shatseva T, Hajian T, Al-Awar R, Dick JE, Vedadi M, Brown PJ, Arrowsmith CH, Bradner JE, Schapira M: Catalytic site remodelling of the DOT1L methyltransferase by selective inhibitors. Nature communications 2012, 3:1288.

[9] Verma SK, Tian X, LaFrance LV, Duquenne C, Suarez DP, Newlander KA, Romeril SP, Burgess JL, Grant SW, Brackley JA, Graves AP, Scherzer DA, Shu A, Thompson C, Ott HM, Aller GS, Machutta CA, Diaz E, Jiang Y, Johnson NW, Knight SD, Kruger RG, McCabe MT, Dhanak D, Tummino PJ, Creasy CL, Miller WH: Identification of Potent, Selective, Cell-Active Inhibitors of the Histone Lysine Methyltransferase EZH2. ACS medicinal chemistry letters 2012, 3:1091-6.

[10] Barsyte-Lovejoy D, Li F, Oudhoff MJ, Tatlock JH, Dong A, Zeng H, Wu H, Freeman SA, Schapira M, Senisterra GA, Kuznetsova E, Marcellus R, Allali-Hassani A, Kennedy S, Lambert JP, Couzens AL, Aman A, Gingras AC, Al-Awar R, Fish PV, Gerstenberger BS, Roberts L, Benn CL, Grimley RL, Braam MJ, Rossi FM, Sudol M, Brown PJ, Bunnage ME, Owen DR, Zaph C, Vedadi M, Arrowsmith CH: (R)-PFI-2 is a potent and selective inhibitor of SETD7 methyltransferase activity in cells. Proceedings of the National Academy of Sciences of the United States of America 2014, 111:12853-8.

[11] Konze KD, Ma A, Li F, Barsyte-Lovejoy D, Parton T, Macnevin CJ, Liu F, Gao C, Huang XP, Kuznetsova E, Rougie M, Jiang A, Pattenden SG, Norris JL, James LI, Roth BL, Brown PJ, Frye SV, Arrowsmith CH, Hahn KM, Wang GG, Vedadi M, Jin J: An orally bioavailable chemical probe of the Lysine Methyltransferases EZH2 and EZH1. ACS chemical biology 2013, 8:1324-34.

[12] Rappold I, Iwabuchi K, Date T, Chen J: Tumor suppressor p53 binding protein 1 (53BP1) is involved in DNA damage-signaling pathways. The Journal of cell biology 2001, 153:613-20.
